# Effect of drought on photosynthesis, total antioxidant capacity, bioactive component accumulation, and the transcriptome of *Atractylodes lancea*

**DOI:** 10.1186/s12870-021-03048-9

**Published:** 2021-06-25

**Authors:** Aqin Zhang, Mengxue Liu, Wei Gu, Ziyun Chen, Yuchen Gu, Lingfeng Pei, Rong Tian

**Affiliations:** 1 School of Pharmacy, Nanjing University of Chinese Medicine, Nanjing, 210000 China; 2 College of Hanlin, Nanjing University of Chinese Medicine, Taizhou, 225300 China; 3 Jiangsu Collaborative Innovation Center of Chinese Medicinal Resources Industrialization, Nanjing University of Chinese Medicine, Nanjing, 210000 China

**Keywords:** *Atractylodes lancea* (Thunb.) DC, RNAseq, Photosynthetic rate, Antioxidative enzyme, Sesquiterpene, Gene expression, Quality formation

## Abstract

**Background:**

*Atractylodes lancea* (Thunb.) DC, a medicinal herb belonging to the Asteraceae family, often faces severe drought stress during its growth. Until now, there has been no research on the effect of drought stress on the quality formation of *A. lancea*. Therefore, the present study aimed to study the effects of drought stress on *A. lancea* through physical and chemical analysis, and to reveal the related molecular mechanisms via transcriptome analysis.

**Results:**

The photosynthesis was markedly inhibited under drought stress. There were alterations to photosynthetic parameters (Pn, Gs, Ci) and chlorophyll fluorescence (Fv/Fm, NPQ), and the chlorophyll content decreased. Twenty genes encoding important regulatory enzymes in light and dark reactions, including the Rubisco gene of the Calvin cycle, were significantly downregulated. After exposure to drought stress for more than 4 days, the activities of four antioxidative enzymes (SOD, POD CAT and APX) began to decrease and continued to decrease with longer stress exposure. Meanwhile, most of the genes encoding antioxidative enzymes were downregulated significantly. The downregulation of 21 genes related to the respiratory electron transport chain indicated that the blocked electron transfer accelerated excessive ROS*.* The MDA content was significantly elevated. The above data showed that 15 days of drought stress caused serious oxidative damage to *A. lancea*. Drought stress not only reduced the size and dry weight of *A. lancea*, but also lowered the amount of total volatile oil and the content of the main bioactive components. The total volatile oil and atractylodin content decreased slightly, whereas the content of atractylon and β-eudesmol decreased significantly. Moreover, ten significantly downregulated genes encoding sesquiterpene synthase were mainly expressed in rhizomes.

**Conclusions:**

After exposed to drought stress, the process of assimilation was affected by the destruction of photosynthesis; stress tolerance was impaired because of the inhibition of the antioxidative enzyme system; and bioactive component biosynthesis was hindered by the downregulation of sesquiterpene synthase-related gene expression. All these had negative impacts on the quality formation of *A. lancea* under drought stress.

**Supplementary Information:**

The online version contains supplementary material available at 10.1186/s12870-021-03048-9.

## Background

*Atractylodes lancea* (Thunb.) DC, a perennial herb belonging to the *Asteraceae* family, has been listed as an endangered medicinal plant [1]. This medicinal herb has been traditionally used as an important crude drug for the treatment of digestive disorders, rheumatic diseases, night blindness, and influenza for a long time [2–5]. Besides that, it has been well proved that the active components from *A. lancea* exert great anti-cancer effect, especially in treatment of cholangiocarcinoma and gastric cancer [6–9]. Recently, it is interesting to find that *A. lancea* is frequently used in the treatment of Coronavirus Disease 2019 (COVID-19) and ranks first among the key Chinese herbal medicines for national epidemic prevention and control in China [10, 11]. So, there is an increasing demand of *A. lance* with high quality. The main production areas in China (Jiangsu, Hubei, and Henan) have a temperate or subtropical climate, with aridity and little rain in spring and high temperature in summer; therefore, *A. lancea* often faces severe drought stress during its growth [12]. In addition, because of the scarcity of agricultural irrigation resources [13], artificial irrigation cannot be widely implemented to alleviate drought stress of *A. lancea*. Therefore, drought has become the main limiting factor affecting the quality formation of *A. lancea*.

The quality formation of medicinal plants is directly or indirectly influenced by the physiological state of plants [14]. Generally, plants with better physiological characteristics, such as more efficient photosynthesis and higher stress tolerance, can form better quality than plants with poor physiological traits [15, 16].

As the most basic and complex physiological process, photosynthesis is critical to all green plants. It is well established that drought stress can considerably influence plants’ photosynthetic physiology by yellowing leaves, closing stomata, and weakening photosynthesis [17, 18]. The decreased photosynthetic capacity due to drought stress not only hampers the plant growth but also is directly/indirectly associated with the reduction of yield and quality in medicinal plants. Therefore, photosynthesis is a crucial and widely used physiological indicator to assess the influence of drought stress on the quality formation of medicinal plants [19]. However, there are still no reports about the photosynthetic response of *A. lancea* to drought stress*.* This hinder the understanding of the influence of drought stress on the quality formation of *A. lancea*.

It is generally believed that drought stress can induce excessive production of reactive oxygen species (ROS) which often disrupt the growth and quality formation of plants [20]. Antioxidative enzyme in plants is the major defense system to scavenge ROS for maintaining the normal physiological state of plants [21]. Thus, the high level of antioxidative enzyme activity is important for plants to tolerate stress for keeping good growth state and forming good quality. Generally, when plants were subjected to drought stress, antioxidative enzymes’ activities increased initially, but decreased with elevated drought stress level [22]. Zhou et al. [23] and Dai et al. [24] reported that short-term drought stress could induce the enhanced activities of antioxidant enzymes in *A. lancea* seedlings to reduce oxidative damage. This indicated that drought stress could affect the antioxidative enzyme activities of *A. lancea.* But, the plant material used in Zhou’s and Dai’s researches were *A. lancea* seedlings. So, it is essential to analysis the alteration of antioxidative enzyme activity in the adulted *A. lancea* exposed to drought stress for understanding of the influence of drought stress on the quality formation of *A. lancea*.

The oil content of *A. lancea* reaches about 2.02% to 4.06% [25]. The volatile oil, mainly composed of sesquiterpenes, is the main active substance of *A. lancea* and has liver-protective, analgesic, anti-viral, and gastrointestinal motility activities [2, 26, 27]. High levels of atractylon, β-eudesmol, and atractylodin are present in the volatile oil, and their levels are commonly used as indices to evaluate the quality of *A. lancea* [28]. Sesquiterpenes are mainly synthesized by the mevalonate (MVA) pathway in plants [29]. The universal precursor, farnesyl diphosphate (FPP), can be converted into different sesquiterpene skeletons by different sesquiterpene synthases (SSs) [30]. Previous studies showed that drought stress changed the content of sesquiterpenes in *Ocimum basilicum L.* by regulating the expression of key genes encoding enzymes of terpene biosynthesis [31]. Until now, some researches on the biosynthetic pathway of sesquiterpenes in *A. lancea* have been reported [32, 33], but, the effect of drought on accumulation and biosynthesis of sesquiterpenes in *A. lancea* is still unclear. Thus, it is essential to determine the accumulation of main bioactive component and to analysis expression level of genes related to sesquiterpene biosynthesis in *A. lancea* under drought stress. This will help us to understand the influence of drought stress on the quality formation of *A. lancea*.

The main research content of the present study was: 1. To establish a transcriptome database of *A. lancea* leaves and rhizomes under normal water management and under drought stress using DNA sequencing, and to verify the accuracy of the data using quantitative real-time reverse transcription PCR (qRT-PCR); 2. To study the effects of drought stress on the photosynthetic physiology of *A. lancea* by measuring chlorophyll content, photosynthesis parameters, and chlorophyll fluorescence (ChlF), and to analyze the expression levels of genes encoding important regulatory enzymes in photosynthesis; 3. To study the antioxidant ability of *A. lancea* under drought by measuring the activity of antioxidative enzymes and analyzing the expression levels of genes associated with antioxidative enzymes and 4. To study the effect of drought stress on the yield and quality of *A. lancea* by determining the dry weight and the content of bioactive components and analyzing the expression levels of genes related to bioactive components’ biosynthesis. Through the achievement of the above research content, we hope to provide a detailed understanding of the influence of drought stress on the quality formation of *A. lancea*. This will not only benefits for developing management strategy of *A. lancea* to minimize the quality reduction induced by drought, but also contributes to future drought tolerance breeding in *A. lancea*.

## Results

### Transcriptomic profiles, differentially expressed genes (DEGs) clustering analysis, and qRT-PCR validation

**Fig. 1:**
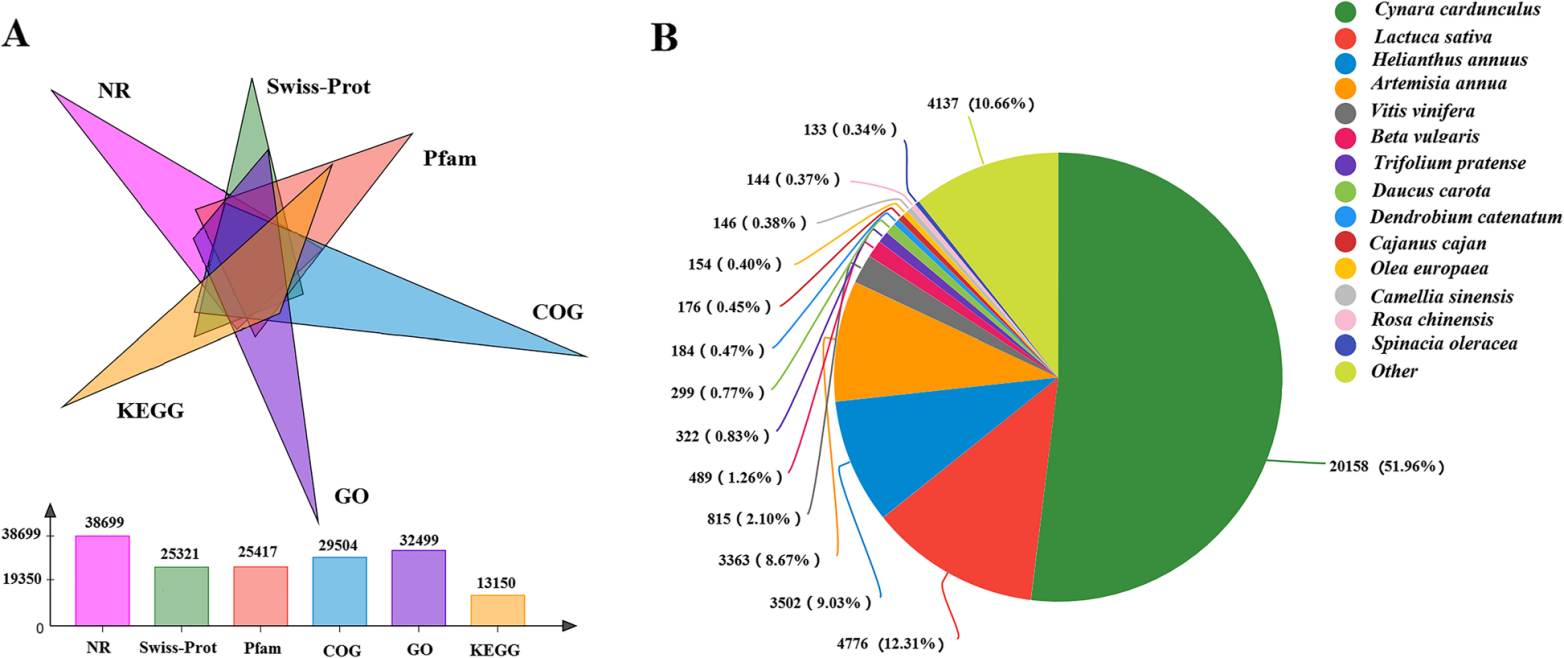
Number of unigenes annotated in the six databases (**A**) and the species annotation results based on NR database (**B**) in *A. lancea*

A total of 351,123,566 high-quality clean reads with a Q20 ≥ 97.86% and a GC base ratio between 44.84% and 46.01% (Table S1) were generated. After assembling the clean reads, 82,677 unigenes and 14,0145 transcripts were obtained (Table S2). The detailed annotation results of the 82,677 unigenes were shown in Fig. 1A, and the top four similar species were *Cynara cardunculus* (51.96%), *Lactuca sativa* (12.31%), *Helianthus annuus* (9.03%), and *Artemisia annua* (8.67%) (Fig. 1B). These four species all belong to the Asteraceae family, as does *A. lancea*.

**Fig. 2:**
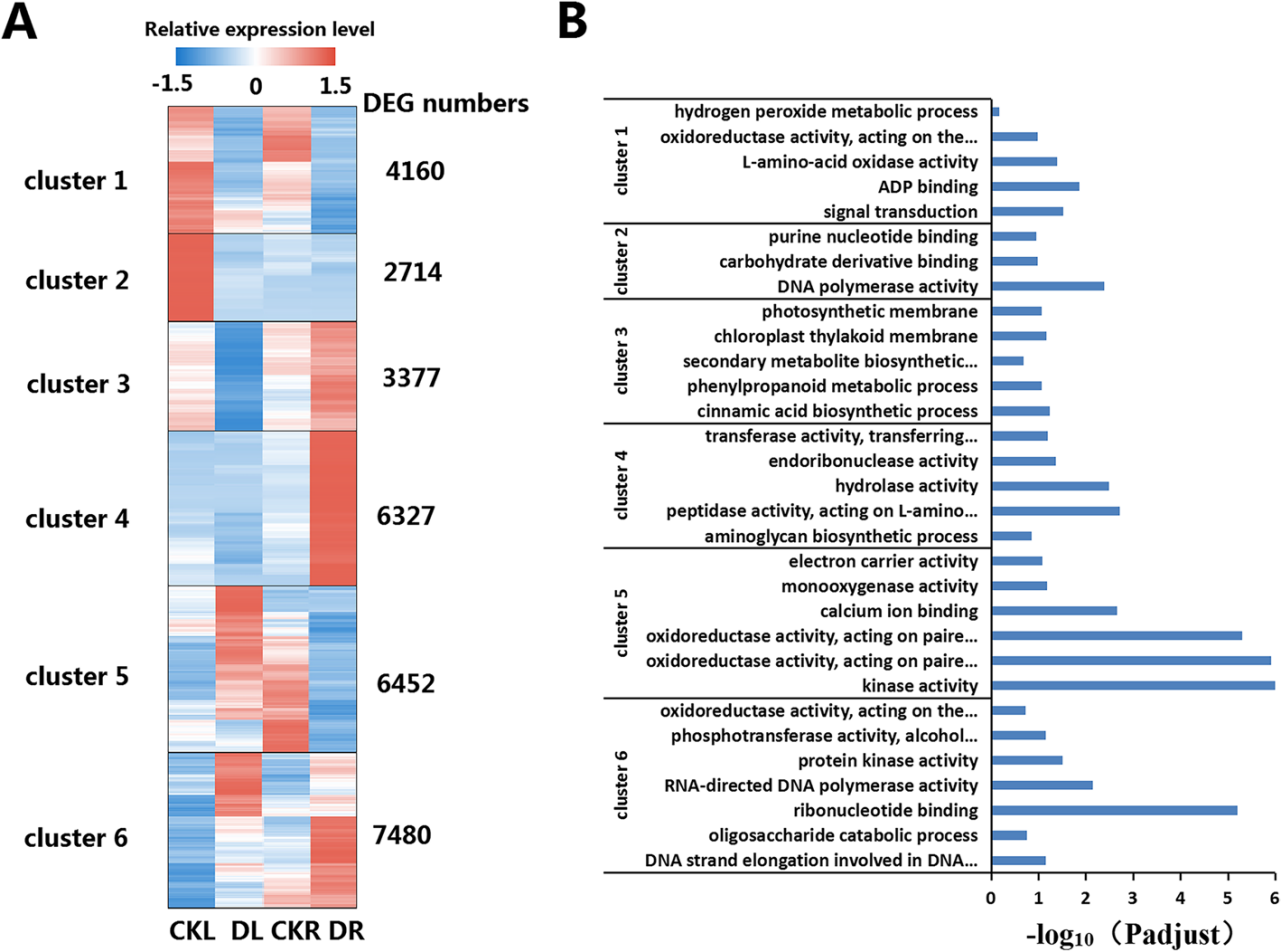
The cluster analysis (**A**) and GO functional enrichment (**B**) of DEGs in *A. lancea*

Using p-adjust < 0.001 and |log2FC|≥ 1, 30,510 DEGs were obtained between drought treatment (DT) group and control (CK) group. Using cluster analysis, these 30,510 DEGs could be divided into six clusters according to their pattern of gene expression. Cluster 1 contained 4160 DEGs that were downregulated in both leaves and rhizomes. Cluster 2 comprised 2714 genes whose expression was downregulated in leaves, but was not significantly different in rhizomes. Cluster 3 contained 3377 genes that were downregulated in leaves and upregulated in rhizomes. Cluster 4 contained 6452 DEGs whose expression was downregulated in rhizomes but was not significantly different in leaves. Cluster 5 contained 6452 DEGs that were upregulated in leaves and downregulated in rhizomes. Cluster 6 contained 7480 genes that were upregulated genes in both leaves and rhizomes (Fig. 2A). GO functional enrichment analysis were performed on the DEGs in the six clusters (Fig. 2B). It is interesting that many DEGs in cluster 1 and cluster 5 were enriched in GO terms associated with redox processes, such as "oxidoreductase activity" (GO:0,016,901), "hydrogen peroxide metabolic process" "(GO:0,042,743), and "monooxygenase activity" (GO:0,004,497). In addition, DEGs in cluster 3 were mainly enriched in GO terms related to photosynthesis, such as "chloroplast thylakoid membrane" (GO:0,009,535) and "photosynthetic membrane" (GO:0,034,357), and these genes are all downregulated in leaves, suggesting that the photosynthesis of *A. lancea* was inhibited by drought stress.

These 30,510 DEGs were also associated with 133 KEGG pathways (Figure S1), among which 100 pathways belonged to metabolism, accounting for the majority of DEGs. It is worth noting that three pathways were related to photosynthesis and five pathways were associated with terpenoid biosynthesis (Table 1).

**Table 1:** Pathways related to photosynthesis and terpenoid biosynthesis

pathway ID	Description	Number of DEGs
map00195	Photosynthesis	18
map00196	Photosynthesis—antenna proteins	6
map00710	Carbon fixation in photosynthetic organisms	51
map00909	Sesquiterpenoid and triterpenoid biosynthesis	26
map00900	Terpenoid backbone biosynthesis	27
map00904	Diterpenoid biosynthesis	14
map00902	Monoterpenoid biosynthesis	25
map01062	Biosynthesis of terpenoids and steroids	1

The expression levels of five selected genes (*DN15090_c0_g1*, *DN474_c1_g1*, *DN18330_c1_g1*, *DN47389_c0_g1*, and *DN54795_c0_g2*) calculated using qRT-PCR were listed in Table S3. The gene expression trends were consistent with those in the transcriptome sequencing data, confirming that the transcriptome-based DEG results were reliable to identify drought-responsive genes in the present study.

### Changes in the photosynthesis system and associated gene expression analysis

**Fig. 3:**
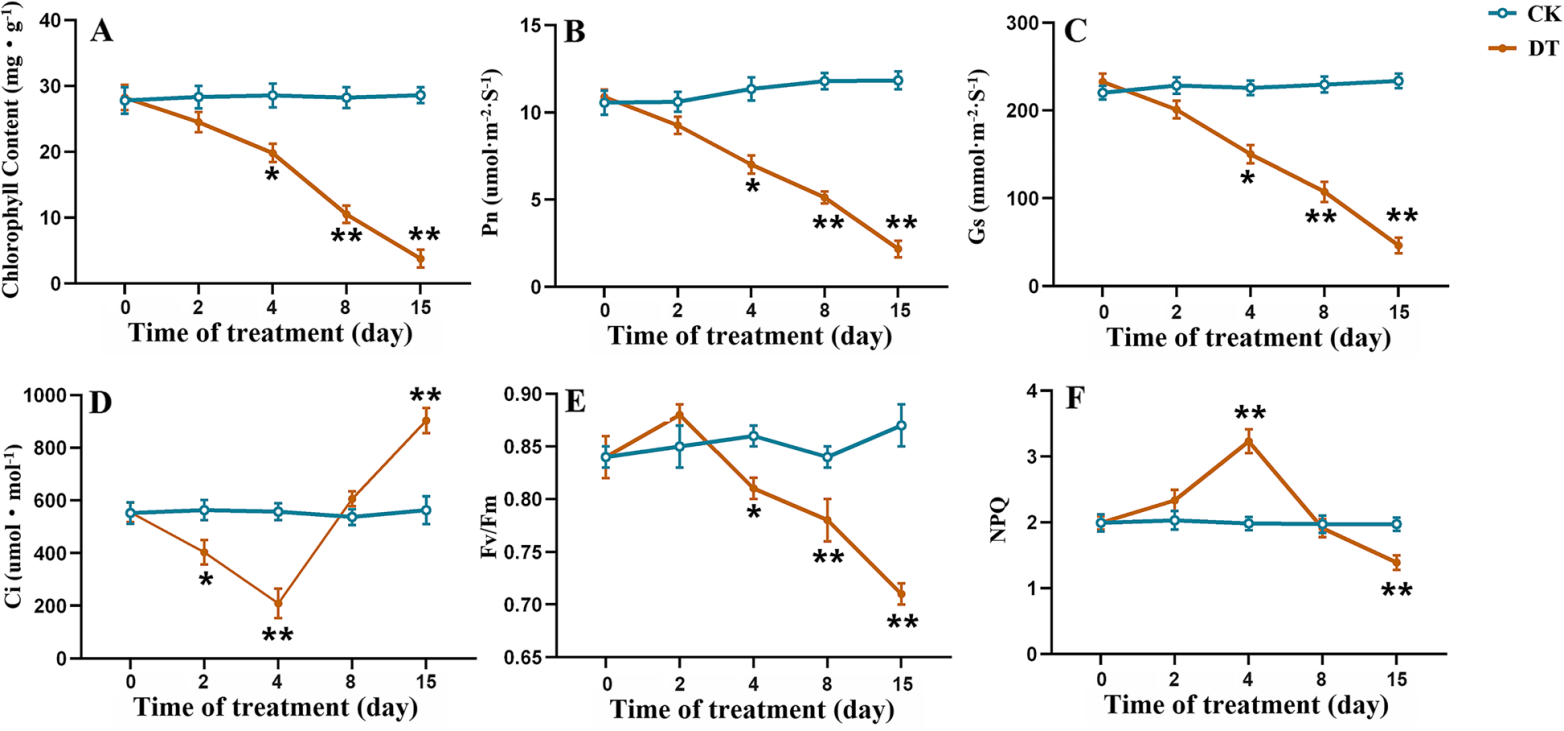
Chlorophyll content (**A**), Pn (**B**), Gs (**C**), Ci (**D**), Fv/Fm (**E**), and NPQ (**F**) of *A. lancea* under drought stress. The values are presented as the means ± SD. One asterisk and two asterisks indicate significantly different values in DT compared to CK at p ≤ 0.05 and p ≤ 0.01, respectively

Under drought stress, all of the measured photosynthesis parameters of *A. lancea* showed obvious alterations. As the duration of drought stress treatment increased, the chlorophyll (CHL) content, net photosynthesis (Pn) and stomatal conductance (Gs) of *A. lancea* all showed continuous downward trends (Fig. 3A-3C). On the 15th of treatment, the values of CHL, Pn and Gs in the DT group were significant smaller than those in the CK group. The intercellular carbon dioxide concentration (Ci) of *A. lancea* started to decrease within 4 days of drought treatment and then began to increase from the 5th day of drought stress treatment, leading to a higher final Ci in the DT group (Fig. 3D). As for chlorophyll fluorescence, drought stress caused a significant reduction in the potential yield of the photochemical reaction of PsII (Fv/Fm) and the longer the drought stress, the greater the reduction (Fig. 3E). The value of non-photochemical quenching (NPQ) also altered significantly as the prolonged drought treatment, increased on the first 4 days, and then decreased from the 5th day (Fig. 3F).

**Fig. 4:**
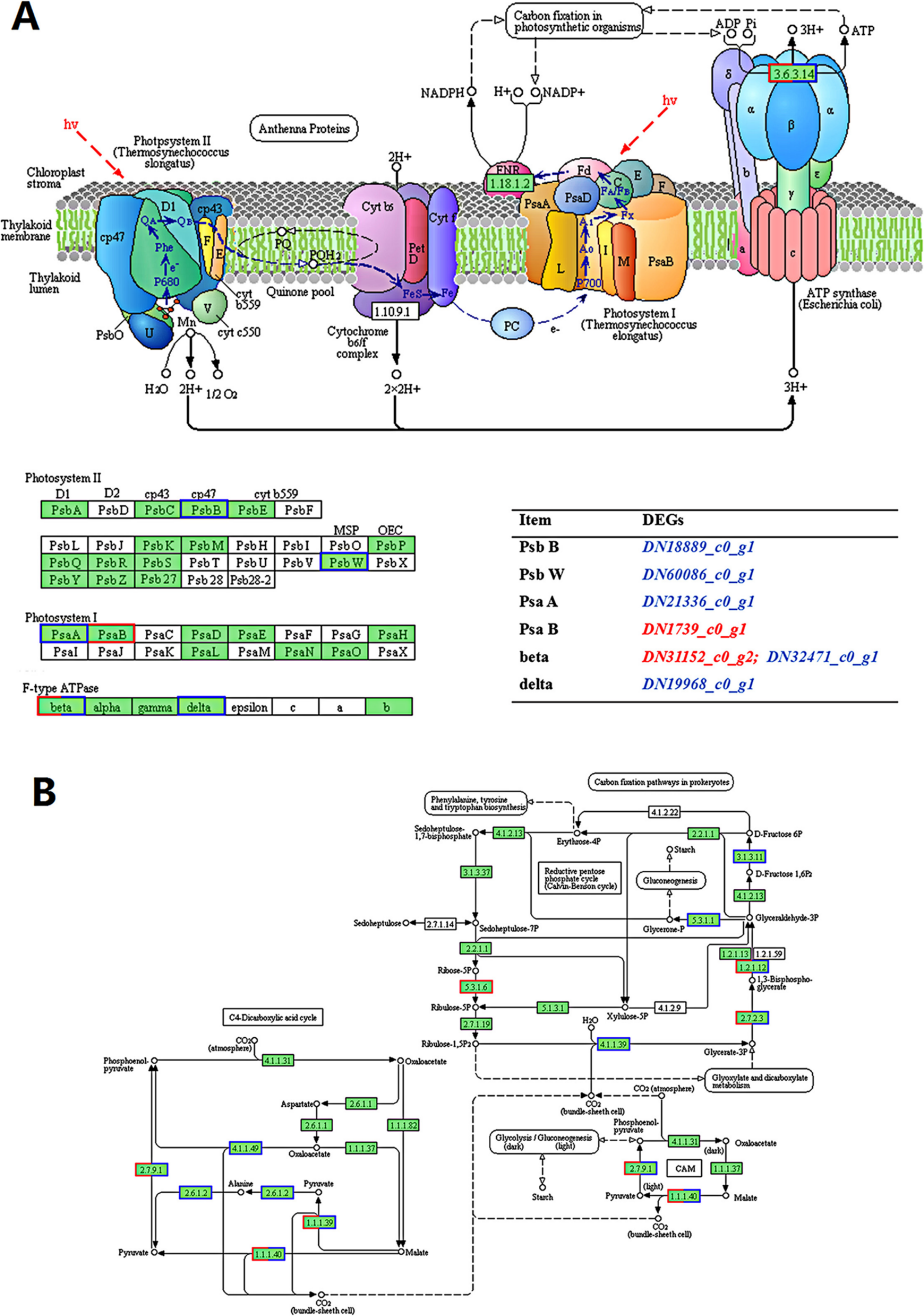
DEGs involved in light reaction (**A**) and dark reaction (**B**) of photosynthesis

To gain a deeper understanding of the mechanisms underlying the reduced photosynthesis of *A. lancea* under drought stress, 31 DEGs encoding key enzymes participating in the light and dark reactions of photosynthesis were identified in leaves. Among these 31 DEGs, 11 were upregulated and 20 were downregulated. In total, among seven DEGs involved in the light reaction (Fig. 4A), *DN31152_c0_g2* (F-type H^+^/Na^+^ ATP synthase gene) and *DN1739_c0_g1* (photosystem I P700 apoprotein A1gene) were upregulated. The other five downregulated DEGs associated with the light reaction were *DN21336_c0_g1* (photosystem I P700 apoprotein A1 gene), *DN19968_c0_g1* and *DN32471_c0_g1* (F-type H^+^/Na^+^ ATP synthase gene), *DN18889_c0_g1* (photosystem II CP47 reaction center protein gene), and *DN60086_c0_g1* (photosystem II reaction center W protein gene). Twenty-four DEGs were involved in dark reaction, among which 9 DEGs were upregulated and 15 were downregulated (Fig. 4B). The upregulated 9 DEGs included five malate dehydrogenase genes (*DN7011_c0_g1*, *DN18398_c0_g1*, *DN48988_c0_g1*, *DN69445_c0_g1* and *DN39038_c0_g1*), a glyceraldehyde-3-phosphate dehydrogenase gene (*DN1992_c4_g1*), a ribose-5-phosphate isomerase gene (*DN1163_c0_g2*), a phosphoglycerate kinase gene (*DN34957_c0_g1*) and a pyruvate, phosphate dikinase gene (*DN23841_c0_g1*). The 15 downregulated DEGs included six malate dehydrogenase genes (*DN1618_c0_g2*, *DN45011_c0_g1*, *DN3381_c1_g1*, *DN16280_c0_g1*, *DN61455_c1_g1*, and *DN17392_c0_g1*), two triosephosphate isomerase genes (*DN7162_c3_g1* and *DN24632_c0_g2)*, a ribulose bisphosphate carboxylas*e* gene (*DN1343_c0_g2*), a glyceraldehyde-3-phosphate dehydrogenase gene (*DN4492_c0_g3*), an alanine aminotransferase gene (*DN14431_c0_g1*), a pyruvate, phosphate dikinase gene (*DN4030_c0_g2*), a phosphoglycerate kinase gene (*DN9649_c0_g1*), a phosphoenolpyruvate carboxykinase gene (*DN3731_c0_g1*), and a fructose-1,6-bisphosphatase gene (*DN39571_c0_g2*).

### Changes in the protective enzymes system and associated gene expression analysis

**Fig. 5:**
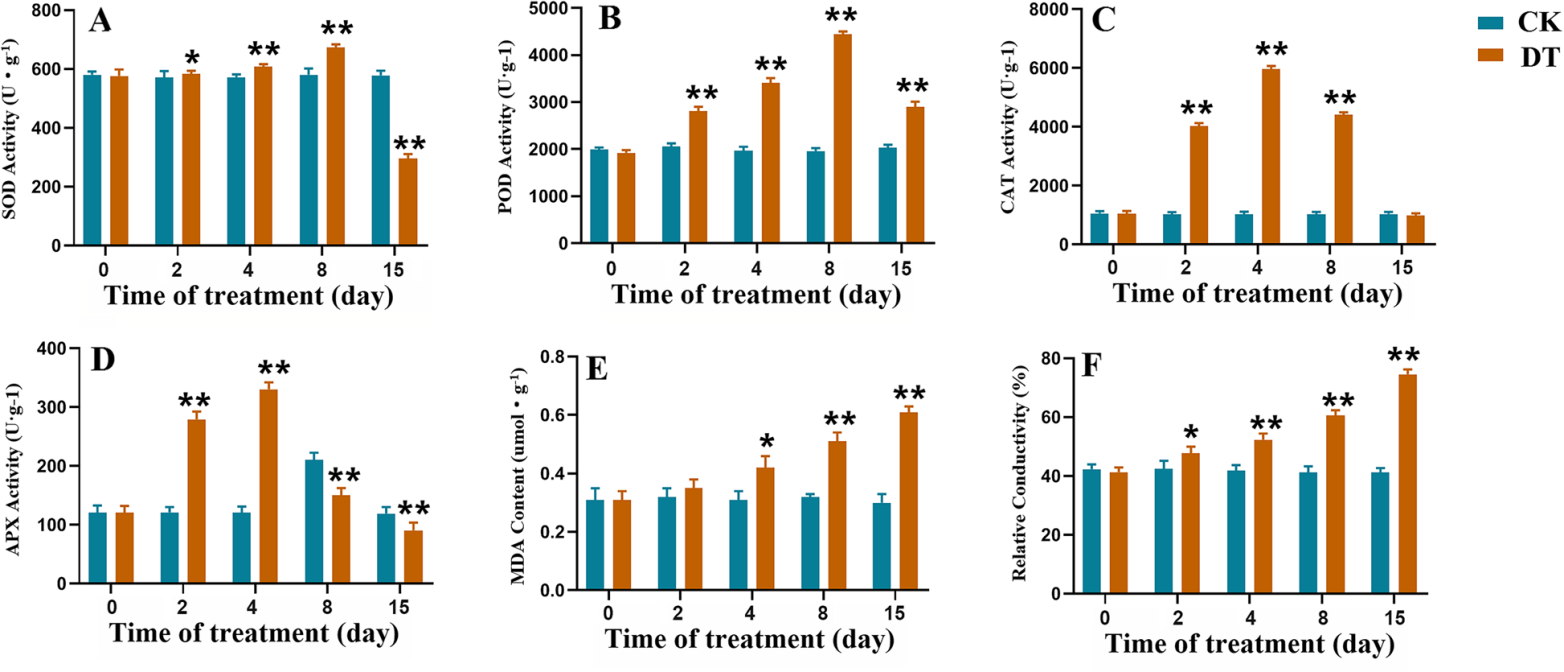
SOD (**A**), POD (**B**), CAT (**C**), APX (**D**) activities, MDA content (**E**) and relative conductivity (**F**) of *A. lance* under drought stress. The values are presented as the means ± SD. One asterisk and two asterisks indicate significantly different values in DT compared to CK at p ≤ 0.05 and p ≤ 0.01, respectively

The activities of SOD, POD, CAT and APX were higher in the DT group than those in the CK group during the early stage of drought stress. For SOD and POD, the early stage of drought stress was 0 to 8 days. For CAT and APX, it was 0 to 4 days. After the early stage, the activities of these enzymes began to decrease. On the 15th day of drought stress, the activity of the four antioxidative enzymes were all different with those in the CK group. The activitives of SOD and APX in the DT group were significantly lower than those in the CK group. The POD activity was significantly higher than that in the CK group and CAT activity is slightly (non-significantly) lower than that in the CK group (Fig. 5A-5D). In addition, the amount of MDA increased rapidly in *A. lancea* under drought stress, while there was no change in MDA levels in the CK group (Fig. 5E). The relative conductivity showed a similar result to the MDA content (Fig. 5F).

The detailed analysis of transcriptome of *A. lancea* under normal water management and under drought stress provided a large number of DEGs encoding antioxidant enzymes. There were five SOD genes (*DN3525_c0_g1*, *DN19179_c0_g3*, *DN19917_c0_g1*, *DN474_c1_g1*, and *DN53562_c0_g1*) and ten CAT genes (*DN5539_c0_g4*, *DN42828_c0_g1*, *DN3870_c0_g1*, *DN49828_c0_g1*, *DN9135_c1_g1*, *DN5359_c0_g1*, *DN3848_c0_g1*, *DN7441_c0_g1*, *DN25688_c0_g1*, and *DN3848_c0_g2*). These 15 DEGs were all downregulated under drought stress. In addition, 18 POD genes were obtained, including six upregulated DEGs (*DN21191_c0_g1*, *DN4672_c0_g2*, *DN51705_c0_g1*, *DN35661_c0_g1*, *DN2622_c1_g1*, and *DN18330_c1_g1*)and 12 downregulated DEGs (*DN838_c0_g1*, *DN68449_c0_g1*, *DN7681_c1_g1*, *DN75393_c0_g2*, *DN9309_c0_g2*, *DN40830_c0_g1*, *DN52474_c0_g4*, *DN42408_c0_g1*, *DN61971_c0_g1*, *DN45395_c0_g1*, *DN19722_c0_g2*, and *DN15530_c0_g3*).

**Fig. 6:**
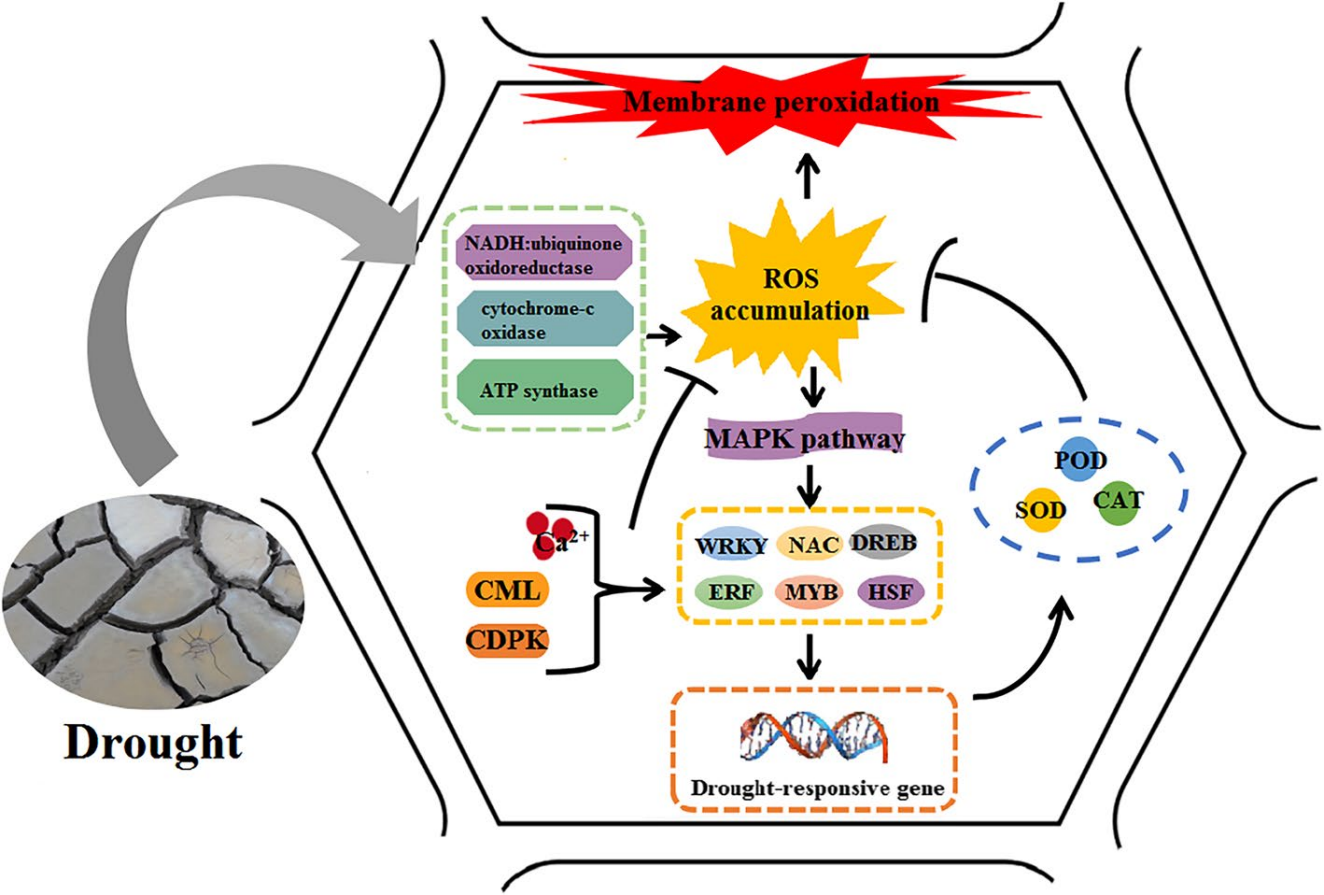
The hypothetical regulatory network about the effect of drought stress on the oxidative enzyme activities of *A. lancea*

It has been reported that ROS can act as second messengers to transduce the signal to transcription factors, thus in turn activate gene expression of antioxdative enzymes. So, we also paid attention to DEGs associated with ROS production and found that 21 downregulated DEGs were related to the respiratory electron transport chain under drought stress (Fig. 6). The 21 DEGs comprised four NADH: ubiquinone oxidoreductase genes (*DN215_c1_g1*, *DN13447_c0_g2*, *DN5922_c2_g2*, and *DN30220_c0_g1*), two cytochrome-c oxidase genes (*DN7184_c0_g1* and *DN26312_c0_g1*), and 15 ATP synthase genes (*DN7129_c0_g1*, *DN1539_c0_g1*, *DN1467_c0_g1*, *DN19968_c0_g1*, *DN5265_c0_g1*, *DN14586_c0_g1*, *DN4776_c0_g1*, *DN32471_c0_g1*, *DN5303_c1_g2*, *DN2129_c1_g1*, *DN2238_c0_g2*, *DN1584_c1_g3*, *DN17811_c0_g1*, *DN27345_c0_g1* and *DN77850_c0_g1*).

### Changes in bioactive component contents and associated gene expression analysis

**Fig. 7:**
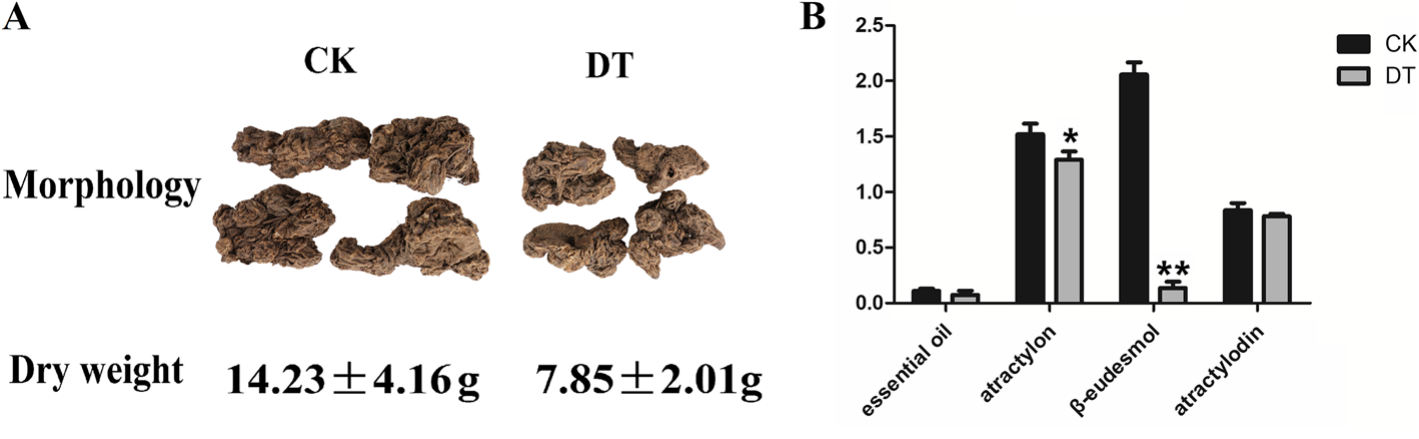
Morphology, dry weight (**A**) and bioactive component content (**B**) of *A. lancea* under drought stress. The determination of each component was performed for three times. One asterisk represents a significant difference of p < 0.05 and two asterisks represent a significant difference of p ≤ 0.01

The rhizomes of *A. lancea*, sampled after 15 days of drought stress treatment, were used as the experimental materials for quality assessment. Morphologically, the rhizomes of *A. lancea* from the DT group were smaller than those from the CK group (Fig. 7A). Moreover, drought stress treatment led to a reduction in the dry weight of *A. lancea* rhizomes. The average dry weight of the rhizomes from the DT group was 7.85 ± 2.01 g, while the average dry weight of the rhizomes from the CK group was 14.23 ± 4.16 g. The proportion of essential oil also showed difference between the two groups. The essential oil content of *A. lancea* in the CK and DTgroup was 0.111 ml/g and 0.096 ml/g, respectively. In addition, the content of three bioactive components (atractylon, β-eudesmol, and atractylodin) all showed decreasing trends in *A. lancea* in the DT group compared with that in the CK group (Fig. 7B). Importantly, the amount of atractylon decreased significantly (P < 0.05). Notably, a sharp reduction (P < 0.01) was observed in the content of β-eudesmol.

**Fig. 8:**
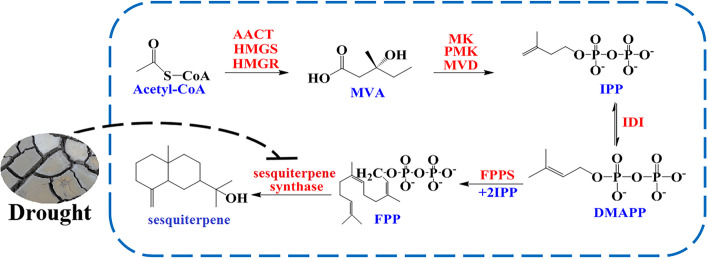
The effect of drought stress on the biosynthesis pathway of sesquiterpene in *A. lancea*

We next turned our attention to DEGs associated with “terpenoid backbone biosynthesis” (map00900) and “sesquiterpenoid and triterpenoid biosynthesis” (map00909) and identified 18 DEGs associated with sesquiterpenoid biosynthesis. Among them, eight DEGs were related to the MVA pathway, including two hydroxymethylglutaryl-CoA synthase genes (*DN8392_c0_g1* and *DN2865_c0_g3*), four hydroxymethylglutaryl-CoA reductase genes (*DN48911_c0_g2*, *DN1889_c2_g2*, *DN86031_c0_g1*, and *DN16497_c0_g1*), and two diphosphomevalonate decarboxylase genes (*DN79187_c0_g1* and *DN23531_c0_g1*). Under drought stress, some of these 8 genes were upregulated and some were downregulated, i.e., they did not show a consistent pattern. In addition, 10 DEGs associated with sesquiterpene synthase were obtained. Among them, seven (*DN56449_c0_g2*, *DN2689_c0_g2*, *DN1597_c0_g1*, *DN54795_c0_g2*, *DN18787_c0_g2*, *DN1159_c0_g1*, and *DN3448_c0_g2*) were significantly downregulated (Fig. 8) and three (*DN18629_c0_g2, DN18787_c0_g1*, and *DN85030_c0_g1*) were upregulated. These sesquiterpene synthase genes were mainly differentially expressed in rhizomes (n = 8) with only two expressed in leaves.

## Discussion

The quality formation of medicinal plant is an extreme complex process which was mainly determined by two aspects: heredity and environment [34]. Certainly, suitable environment is quite important for the quality formation of medicinal plants [35]. However, with the deterioration of the global climate, encountering drought stresses has been more common and frequent during medicinal plants’ growth. Thus, drought has become a crucial limiting factor that influence the quality formation of medicinal plants [36, 37]. In present study, the effect of drought stress on the quality formation of *A. lancea* was studied by analyzing photosynthesis, antioxidative enzyme activity and bioactive component accumulation.

Photosynthesis is particularly sensitive to drought stress and often is inhibited because of stomata closure induced reduced CO_2_ supply and further metabolic impairment by drought stress [38, 39]. Generally, the total dry matter production of a plant is equal to its net photosynthesis [40]. So, drought stress causes a reduction of photosynthesis rates in medicinal plants, leading to low yields of medicinal plants. In present study, the significant decrease of Pn in *A. lancea* from the DT group confirmed that drought stress lasted for 15 days caused obvious photosynthesis inhibition in *A. lancea*. This result was consistent with previous studies [41, 42]. The continuous declined Gs indicated that limitations of CO_2_ uptake caused by stomata closure was responsible for photosynthesis inhibition [43]. With prolonged drought stress, Ci began to increase, suggesting that metabolic impairment induced by drought became another main limiting factor. Rubisco (ribulose bisphosphate carboxylase) is the key enzyme participated in the first stage of the Calvin cycle by catalyzing ribulose-1,5-bisphosphate and CO_2_ to form two molecules of 3 phosphoglyceric acid (3-PGA) [44]. In the present study, A Rubisco gene (*DN1343_c0_g2)* was significantly downregulated under drought stress, indicating that drought stress caused metabolic impairment in *A. lancea* by inhibiting the carboxylation reaction of CO_2_. The rate of photosynthesis also depends on the regeneration of RuBP [45]. Fructose-1,6-bisphosphatase (FBP) and triosephosphate isomerase (TPI) can catalyze glyceraldehyde-3-phosphate (GAP) through a series of transformations to reform RuBP [46, 47]. A FBP gene (*DN39571_c0_g2*) and two TPI genes (*DN7162_c3_g1* and *DN24632_c0_g2*) were significantly downregulated, indicated that drought stress could also cause metabolic impairment in *A. lancea* by hindering the regeneration of RuBP.

The Fv/Fm ration represent the potential yield of the photochemical center of photosystem II (PSII) [48]. The value of Fv/Fm ration has been reported to stay around 0.83 among many different plant species under normal growth conditions [49]. When the plant was exposed to drought stress, Fv/Fm is particularly sensitive and its value decreased greatly [50]. Thus, Fv/Fm has already become an important parameter to judge the physiological state of plants under drought stress. In present study, the Fv/Fm value of *A. lancea* in the CK group was kept at a relative stable level, while the Fv/Fm in the DT group showed a significantly reduction. This demonstrated that the efficiency of light energy conversion and electron transfer activities were all markedly inhibited under drought stress. The characteristic decrease of Fv/Fm induced by drought stress was consistent with previous studies [51, 52]. The reduction in Fv/Fm is often associated with an increase in the efficiency of non-photochemical quenching (NPQ) [53]. Under drought stress, the significantly increased NPQ showed that excessive irradiation could be dissipated into heat to protect *A. lancea* from drought-induced damage. The observed reduction in efficiency of PSII was concomitant with the down-regulation of genes belonging to the PSII category induced by drought stress, evidencing that the reduced efficiency of PSII was the other main limiting factor of photosynthesis inhibition in *A. lancea* under drought stress.

Combined with the lower dry weight of *A. lancea* under drought stress, we inferred that drought inhibited photosynthesis of *A. lancea* by closing stomata, reducing the CHL content, limiting the efficiency of PSII and inducing metabolic impairment, which led to a yield decline, thereby affecting the quality formation of *A. lancea*.

Drought stress inevitably causes excessive production of ROS, which can oxidize biofilms, form lipid peroxidation products, and damage cells [54]. Antioxidative enzymes form the first line of defense against ROS and have been reported to contribute directly or indirectly in drought tolerance of many plants, such as rice [55], alfalfa [56] and canola [57]. Adebayo (2015) stated that sustained yields in maize under drought stress were directly related to better antioxidant activities [58]. In present study, the activities of SOD, POD, CAT and APX were higher in the DT group than those in the CK group during the early stage of drought stress. This was consistent with Zhou’s and Dai’s researches. This implied that during the early stage of drought stress, ROS acted as second messengers involved in the stress signal transduction pathway to activate antioxidative enzyme activity. Like other plants, the induction of antioxidant enzyme activity is an adaptation strategy which *A. lancea* use to overcome oxidative stresses. The balance between ROS production and activities of antioxidative enzymes determined whether oxidative signaling and/or damage would occur [59]. As the duration of drought treatment increased, the activities of all four antioxdative enzymes decreased. On the 15th day, except for POD, the activities of SOD, CAT and APX were lower compared with those in the CK group. And gene expression analysis identified 35 antioxidant enzyme genes and 26 of them were significantly downregulated under 15 days’ drought stress. This may reflect the low ROS scavenging capacity and increased damage in *A. lancea* under 15 days’ drought stress. The decrease of antioxidant enzyme activity under progressive drought stress also has been reported by many previous studies. In addition, 21 significantly downregulated genes related to the respiratory electron transport chain in *A. lancea* under drought stress, suggested that the blocked electron transport would accelerate the accumulation of ROS [60]. So, it could be concluded that the level of ROS induced by 15 days of drought stress in *A. lancea* was beyond the adjustable range of the plant itself, and the antioxidative enzyme system could no longer remove the excessive ROS. The markedly elevated level of MDA and relative conductivity under drought stress also evidenced the severe oxidative damage to *A. lancea* caused by drought stress. Taken together, our data suggested that the significant decline of antioxidative enzyme activity was an important factor affecting *A. lancea*’s stress tolerance, thereby affecting the quality formation of *A. lancea.*

The amount of volatile oil and the content of atractylon, β-eudesmol, and atractylodin are often used as indices to evaluate the quality of *A. lancea* [28]. In present study, not only the amount of volatile oil, but also the content of atractylon, β-eudesmol, and atractylodin decreased in *A. lancea* under drought stress. This confirmed that 15 days’ drought stress negatively affected the quality of *A. lancea*. Expression analysis of genes related to sesquiterpene biosynthesis provided 18 DEGs. Although there was not a consistent pattern about the expression regulation of DEGs involved in the upstream of sesquiterpene biosynthesis, seven DEGs ecoding sesquiterpene synthase, a key regulatory enzyme in the downstream of sesquiterpene biosynthesis were significantly downregulated. This suggested that drought stress could reduce the accumulation of sesquiterpene components by reducing the expression levels of associated sesquiterpene synthase genes. Overall, the downregulated gene expression of sesquiterpene synthase and the low amounts of volatile oil, atractylon, β-eudesmol, and atractylodin illustrated that drought stress slowed down the bioactive components’ biosynthesis and accumulation, thereby affecting the quality formation of *A. lancea*.

## Conclusions

Drought stress affected the quality formation of *A. lancea* from the following three aspects. First, genes encoding important regulatory enzymes in dark reaction were downregulated significantly, the efficiency of PSII was limited, thus, photosynthesis was significantly inhibited. Second, gene expression and activities of antioxidative enzymes were mostly downregulated, thus, the stress tolerance of *A. lancea* was impaired. Third, gene expression of key enzymes involved in sesquiterpene biosynthesis were down-regulated, thus, the accumulation of bioactive components was slowed down in *A. lancea*.

## Methods

### Plant materials and drought stress treatment

*A. lancea* was obtained from Maoshan (N31°79′14.98’’, E119°32′19.56’’), Jurong, Jiangsu Province, China and identified as *Atractylodes lancea* (Thunb.) DC by Professor Wei Gu (School of Pharmacy, Nanjing University of Chinese Medicine, China). The voucher was deposited in Department of Chinese Medicine Resources, Nanjing University of Chinese Medicine with ID MCZ-19–001.The *A. lancea* used in the present study were artificially cultivated, so, there was no permission needed for the collection of *A. lancea*. Then the plants were transplanted in the Medicinal Botanical Garden, Nanjing University of Traditional Chinese Medicine (NUTCM). Five *A. lancea* plants were in a plastic pot (top diameter 38 cm, bottom diameter 32 cm, height 35 cm) with approximately 20 kg dry soil. The potted plants were grown in a greenhouse with 25 °C, 60% relative humidity and 14 h of light and 10 h of darkness. Soil taken from the Medicinal Botanical Garden of NUTCM was air-dried naturally and then passed through a 5 mm sieve. The experimental soil was prepared by mixing the air dried soil and farmyard manure evenly in a ratio of 4:1. The detailed information of the prepared experimental soil was as followed: texture, loam; organic matter, 12.74 g/kg; total nitrogen, 2.12 g/kg, total phosphorous, 3.57 g/kg; total potassium, 12.53 g/kg; alkalihydro nitrogen, 63.38 mg/kg; available phosphorus, 20.53 mg/kg; available potassium, 49.86 mg/kg; pH, 6.52. After adaption for two weeks, sixty *A*. *lancea* plants with similar growth rates were randomly divided into two groups: CK group and DT group. Soil water content was measured using a TZS-IIW Soil Moisture Meter (Zhejiang Top Instrument Co., Ltd, Zhejiang, China). The water content was kept at 55% to 65% and 25% to 35% of saturated soil water content in the CK group and the DT group for 15 days, respectively. At 0, 2, 4, 8, and 15 days of the stress treatment, leaves of the two groups were collected and divided into two parts. One part was used for physiological index measurements and the other part was immediately frozen in liquid nitrogen for RNA extraction. In addition, the rhizomes of the two groups were collected at 0, 2, 4, 8, and 15 days and were also divided into two parts. One half of each sample was frozen in liquid nitrogen to be used for RNA extraction, and the other half was oven-dried at 40 °C to a constant weight for quality analysis.

### Illumina sequencing and functional annotation

Leaves and rhizomes of the two groups collected at 15 days were used to isolate total RNA using an RNeasy Plus Mini Kit (#74,134; Qiagen, Hilden, Germany). The cDNA was synthesized using the fragmented mRNA as a template. PCR amplification was then performed, and then high-throughput sequencing was performed on a Illumina HiSeq TM 4000 instrument by Shanghai Megi Biomedical Technology Co., Ltd (Shanghai, China). Fastx_toolkit_0.0.14 (http://hannonlab.cshl.edu/fastx_toolkit/) was used for quality assessment of the raw sequencing data. After obtaining high-quality clean reads. Trinity_v2.8.5 (https://github.com/trinityrnaseq/trinityrnaseq) was used to assemble the clean reads into transcript sequences, and TransRate (http://hibberdlab.com/transrate) was applied to filter the transcripts. The optimized transcript sequences were stored as FASTQ files under the project accession numbers (SRR8699030, SRR8699031, SRR8699032, and SRR8699033) in the Short Read Archive database of the NCBI.

Gene annotation information was obtained by BlastX searching in the NCBI non-redundant protein database (NR, http://www.ncbi.nlm.nih.gov), and by analysis using the following database resources: Cluster of Orthologous Groups (COG), Swissprot (http://www.expasy.ch/sprot), Kyoto Encyclopedia of Genes and Genomes (KEGG, http://www.genome.jp/kegg), Pfam (http://pfam.xfam.org/), and Gene Ontology (GO, http://www.geneontology.org). The E-value was set to 1E^−5^.

### Gene expression analysis and qRT-PCR validation

Gene expression was calculated using RSEM (http://deweylab.github.io/RSEM/) and normalized by the FPKM (Fragments per Kilobases per Million reads) value. EdgeR_3.24.3 (http://bioconductor.org/packages/stats/bioc/edgeR/) were used to obtain DEGs, and Bonferroni one-step correction was used as a multiple test correction method, and p-adjust < 0.05 and |log2fold-change (FC)|≥ 2 were used as criteria. Fisher's exact test was used for DEG enrichment analysis. When the false discovery rate (FDR) was < 0.05, the GO function item or the KEGG pathway was considered to be significantly enriched for the DEG.

Expression levels of five randomly selected genes (*DN15090_c0_g1*, *DN474_c1_g1*, *DN18330_c1_g1*, *DN47389_c0_g1*, and *DN54795_c0_g2*) were analyzed to validate the accuracy of transcription sequencing results. The qRT-PCR reaction was performed according to the Platinum® SYBR® Green qPCR Supermix UDG real-time PCR kit instructions (Thermo Fisher Scientific, Waltham, MA, USA). The qPCR reaction comprised: Pre-denaturation at 94 °C for 5 min, followed by 40 cycles of denaturation at 94 °C for 30 s, annealing at 62 °C for 30 s, and extension at 72 °C for 40 s. *EF1A1* (encoding EF-1α) was used as an internal reference gene, and the 2^−ΔΔCt^ method was used to calculate the relative expression level of a gene. The experiment was performed three times, and each sample was assessed three times. The specific qPCR primers were designed using Primer Premier 5.0 software (PREMIER Biosoft International,San Francisco, CA, USA) and were supplied by Generay Biotech Co., Ltd. (Shanghai, Chins; Table S4).

### Photosynthesis measurement

An LI-6400XT photosynthesis-fluorescence measurement system was applied to measure photosynthesis and chlorophyll fluorescence parameters. The determination of net photosynthesis (Pn), stomatal conductance (Gs), and the intercellular carbon dioxide concentration (Ci) were carried out between 9:00 and 11:00 in the morning. After being subjected to dark adaptation treatment for 2 h from 12 noon, the plants were subjected to photochemical efficiency measurement to obtain the Fv/Fm (the ratio of variable to maximum fluorescence). Then, after 1 h of light activation, non-photochemical quenching (NPQ) was measured. The content of CHL was determined using Arnon’s method [61].

### Protective enzyme activity

The content of MDA was measured using the thiobarbituric acid method [62]. A conductivity meter, BDS-6300 (Shanghai Yidian Scientific Instrument Co., Ltd. Shanghai, China), was used to determine the relative conductivity. The riboflavin-trinitrotoluene chloride (NBT) method and guaiacol method were applied to detect SOD and POD activity [63]. The activities of CAT and APX were determined using a UV spectrophotometer UV 5200 (Shanghai Metash Instruments Co., Ltd. Shanghai, China) at 240 nm and 290 nm, respectively [62].

### Determination of bioactive components

Essential oil was collected by steam distillation according to the volatile oil extraction method in the 2020 edition of the Chinese Pharmacopoeia [64].

A Waters e2695 series high performance liquid chromatography (HPLC) system (Waters Technology (Shanghai) Co. Ltd., Shanghai, China) was employed to analyze the samples. A Welchrome C_18_ analytical column (250 mm × 4.6 mm, 5 mm; Shanghai Welch Technology Co. Ltd., Shanghai, China) was used and the mobile phase was acetonitrile (A) and 0.1% v/v formic acid solution (B) at a flow rate of 1 mL·min^−1^. The gradient was as follows: 0–5 min, 10% A, 5–6 min, 14% A, 6–22 min, 30% A, 22–27 min, 52% A, 27–55 min, 80% A, 55–65 min, 100% A. The column temperature was 30 °C and the injection volume was 10 µL. The detection wavelength was 340 nm for atractylodin and 203 nm for atractylon and β-eudesmol, respectively. The reference standards of atractylodin, atractylon, and β-eudesmol were purchased from Shanghai Yuanye Bio-Technology Co., Ltd (Shanghai, China).

Four hundred milligrams of rhizome sample powder and 10 mL of methanol were placed together in a brown triangular bottle and weighted precisely. After ultrasonic extraction for 40 min and cooling to room temperature, methanol was added to the mixture to make up for weight loss. Then, the supernatant was passed through a 0.45-μm micro-porous membrane to prepare the sample solution for HPLC analysis.

### Statistical analysis

All data were assessed for significant differences (P < 0.05) using one-way analysis of variance (ANOVA) with Bonferroni correction using SPSS 20.0 software (IBM Corp., Armonk, NY, USA). Each experiment was performed three times independently and all data were presented as the mean and standard deviation (SD). The GraphPad Prism 8 (GraphPad Software, San Diego, CA, USA) was used to construct graphs.

## Supplementary Information

**Additional file 1: TableS1**. Summary of RNA-Seq database from *A.lancea* under drought stress. **Table S2**. The detailed information for assembled unigenes of *A. lancea* under drought stress. **Table S3**. QRT-PCR validation of DEGs from *A. lancea*. **Table S4**. The primer list of DEGs for qRT-PCR validation. **Figure S1**. Functional classification for assembled unigenes of *A. lancea* by KEGG.

## Data Availability

The data was accessible in NCBI with the direct link and login number: https://www.ncbi.nlm.nih.gov/sra/?term=SRR8699030, SRR8699030. https://www.ncbi.nlm.nih.gov/sra/?term=SRR8699031, SRR8699031. https://www.ncbi.nlm.nih.gov/sra/?term=SRR8699032, SRR8699032. https://www.ncbi.nlm.nih.gov/sra/?term=SRR8699033, SRR8699033.

## Abbreviations

SOD: Superoxide dismutase
POD: Peroxidase
CAT: Catalase
APX: Ascorbate peroxidase
COVID-19: Coronavirus Disease 2019
AsA-GSH: Ascorbate–glutathione
MDA: Malondialdehyde
MVA: Mevalonate
FPP: Farnesyl diphosphate
SS: Sesquiterpenes synthase
QRT-PCR: Quantitative real-time reverse transcription PCR
DEG: Differentially expressed gene
DT: Drought treatment
NR: NCBI non redundant protein database
COG: Cluster of Orthologous Groups
KEGG: Kyoto Encyclopedia of Genes and Genomes
GO: Gene Ontology
ROS: Reactive oxygen species
CHL: Chlorophyll
Rubisco: Ribulose bisphosphate carboxylase
Fv/Fm: The potential yield of the photochemical reaction of PsII
NPQ: Non-photochemical quenching
3-PGA: 3-Phosphoglyceric acid
FBP: Fructose-1,6-bisphosphatase
TPI: Triosephosphate isomerase
GAP: Glyceraldehyde-3-phosphate
