# Supplementary material for: Effect of drought on photosynthesis, total antioxidant capacity, bioactive component accumulation, and the transcriptome of Atractylodes lancea
Source: BMC Plant Biol. 2021 Jun 25;21:293. doi: 10.1186/s12870-021-03048-9 (PMC8226357; doi:10.1186/s12870-021-03048-9)
Supplement: Supplementary file 1 — Additional file 1: TableS1. Summary of RNA-Seq database from A.lancea under drought stress. Table S2. The detailed information for assembled unigenes of A. lancea under drought stress. Table S3. QRT-PCR validation of DEGs from A. lancea. Table S4. The primer list of DEGs for qRT-PCR validation. Figure S1. Functional classification for assembled unigenes of A. lancea by KEGG. [file 12870_2021_3048_MOESM1_ESM.zip › Table S1_ESM.docx]

Table S1. Summary of RNA-Seq database from *A. lancea* under drought stress

| Sample | Clean reads | Clean bases | Error rate(%) | Q20(%) | Q30(%) | GC content(%) |
| --- | --- | --- | --- | --- | --- | --- |
| CKL | 90333134 | 13537832626 | 0.0129 | 97.92 | 94.38 | 46.01 |
| CKR | 78305700 | 11733647370 | 0.0127 | 97.96 | 94.54 | 44.84 |
| DL | 95839882 | 14350973498 | 0.0126 | 98.04 | 94.69 | 45.85 |
| DR | 86644850 | 12965038421 | 0.0129 | 97.86 | 94.34 | 44.84 |
