# Supplementary material for: Effect of drought on photosynthesis, total antioxidant capacity, bioactive component accumulation, and the transcriptome of Atractylodes lancea
Source: BMC Plant Biol. 2021 Jun 25;21:293. doi: 10.1186/s12870-021-03048-9 (PMC8226357; doi:10.1186/s12870-021-03048-9)
Supplement: Supplementary file 1 — Additional file 1: TableS1. Summary of RNA-Seq database from A.lancea under drought stress. Table S2. The detailed information for assembled unigenes of A. lancea under drought stress. Table S3. QRT-PCR validation of DEGs from A. lancea. Table S4. The primer list of DEGs for qRT-PCR validation. Figure S1. Functional classification for assembled unigenes of A. lancea by KEGG. [file 12870_2021_3048_MOESM1_ESM.zip › Table S2_ESM.docx]

Table S2. The detailed information for assembled unigenes of *A. lancea* under drought stress

| Type | Unigene | Transcript |
| --- | --- | --- |
| Total number | 82677 | 140145 |
| Total base | 87367454 | 1.62E+08 |
| Largest length (bp) | 14973 | 14973 |
| Smallest length (bp) | 201 | 201 |
| Average length (bp) | 1056.73 | 1158.03 |
| N50 length (bp) | 1549 | 1644 |
| E90N50 length (bp) | 2338 | 2076 |
| Fragment mapped percent(%) | 70.877 | 82.594 |
| GC percent (%) | 40.48 | 40.73 |
| TransRate score | 0.24457 | 0.33419 |
| BUSCO score | C:70.8%[S:67.6%;D:3.2%] | C:70.8%[S:67.6%;D:3.2%] |
