# Supplementary material for: Effect of drought on photosynthesis, total antioxidant capacity, bioactive component accumulation, and the transcriptome of Atractylodes lancea
Source: BMC Plant Biol. 2021 Jun 25;21:293. doi: 10.1186/s12870-021-03048-9 (PMC8226357; doi:10.1186/s12870-021-03048-9)
Supplement: Supplementary file 1 — Additional file 1: TableS1. Summary of RNA-Seq database from A.lancea under drought stress. Table S2. The detailed information for assembled unigenes of A. lancea under drought stress. Table S3. QRT-PCR validation of DEGs from A. lancea. Table S4. The primer list of DEGs for qRT-PCR validation. Figure S1. Functional classification for assembled unigenes of A. lancea by KEGG. [file 12870_2021_3048_MOESM1_ESM.zip › Table S3_ESM.docx]

| **Category** | **Cene** | **Tissue** | **Group** | **Gene expression level** | | | | |
| --- | --- | --- | --- | --- | --- | --- | --- | --- |
|  |  |  |  | **0d** | **2d** | **4d** | **8d** | **15d** |
| Photosynthesis system | *TRINITY_DN15090_c0_g1* | Leaf | CK | 1.00 | 1.04 | 1.03 | 1.11 | 1.06 |
|  |  |  | DT | 1.02 | 1.03 | 0.89 | 0.57 | 0.31 |
| Peroxidation Resistance | *TRINITY_DN474_c1_g1* | Leaf | CK | 1.00 | 1.02 | 0.99 | 0.94 | 1.20 |
|  |  |  | DT | 1.10 | 6.68 | 16.28 | 2.63 | 0.89 |
|  | *TRINITY_DN18330_c1_g1* | leaf | CK | 1.00 | 0.99 | 0.92 | 1.02 | 1.11 |
|  |  |  | DT | 1.11 | 4.73 | 12.42 | 9.38 | 5.02 |
|  | *TRINITY_DN47389_c0_g1* | leaf | CK | 1.00 | 1.11 | 1.96 | 0.93 | 1.38 |
|  |  |  | DT | 1.10 | 4.16 | 12.99 | 3.40 | 2.29 |
| Sesquiterpene Biosynthesis | *TRINITY_DN54795_c0_g2* | rhizome | CK | 1.00 | 0.93 | 1.02 | 1.11 | 1.04 |
|  |  |  | DT | 1.02 | 1.12 | 0.79 | 0.54 | 0.19 |

**Table S****3. QRT-PCR validation of DEGs from *A. lancea***
