# Supplementary material for: Effect of drought on photosynthesis, total antioxidant capacity, bioactive component accumulation, and the transcriptome of Atractylodes lancea
Source: BMC Plant Biol. 2021 Jun 25;21:293. doi: 10.1186/s12870-021-03048-9 (PMC8226357; doi:10.1186/s12870-021-03048-9)
Supplement: Supplementary file 1 — Additional file 1: TableS1. Summary of RNA-Seq database from A.lancea under drought stress. Table S2. The detailed information for assembled unigenes of A. lancea under drought stress. Table S3. QRT-PCR validation of DEGs from A. lancea. Table S4. The primer list of DEGs for qRT-PCR validation. Figure S1. Functional classification for assembled unigenes of A. lancea by KEGG. [file 12870_2021_3048_MOESM1_ESM.zip › Table S4_ESM.docx]

**Table S4 The primer list of DEGs for qRT-PCR validation**

| **Gene** | **Forward** | **Reverse** |
| --- | --- | --- |
|  |  |  |
| *TRINITY_DN15090_c0_g1* | CGATCGATCCGTCGGTACAT | CCTGCCCGCAATCTACATCA |
| *TRINITY_DN474_c1_g1* | CCTATCATGCCCCTGCAGAA | CTCCTCAGAATACCTTGTTGGCT |
| *TRINITY_DN18330_c1_g1* | GAACGTCAATCCAAGCAGCC | TTGAGGAGTGGACGAGGAGT |
| *TRINITY_DN47389_c0_g1* | CCCACGGTGTCATCAATCCA | AGGGTAGCATCCTCACTGGT |
| *TRINITY_DN54795_c0_g2* | AGGATCCTTGGCCTTGATGC | ACGTCCAAATCCTTCCACCA |
| *EF-1α* | ACCAACTGGGTTGACAACTGAAGT | AGCCTCGGTAAGGGCTTCAT |
