# Supplementary figures and images for: Effect of drought on photosynthesis, total antioxidant capacity, bioactive component accumulation, and the transcriptome of Atractylodes lancea
Source: BMC Plant Biol. 2021 Jun 25;21:293. doi: 10.1186/s12870-021-03048-9 (PMC8226357; doi:10.1186/s12870-021-03048-9)

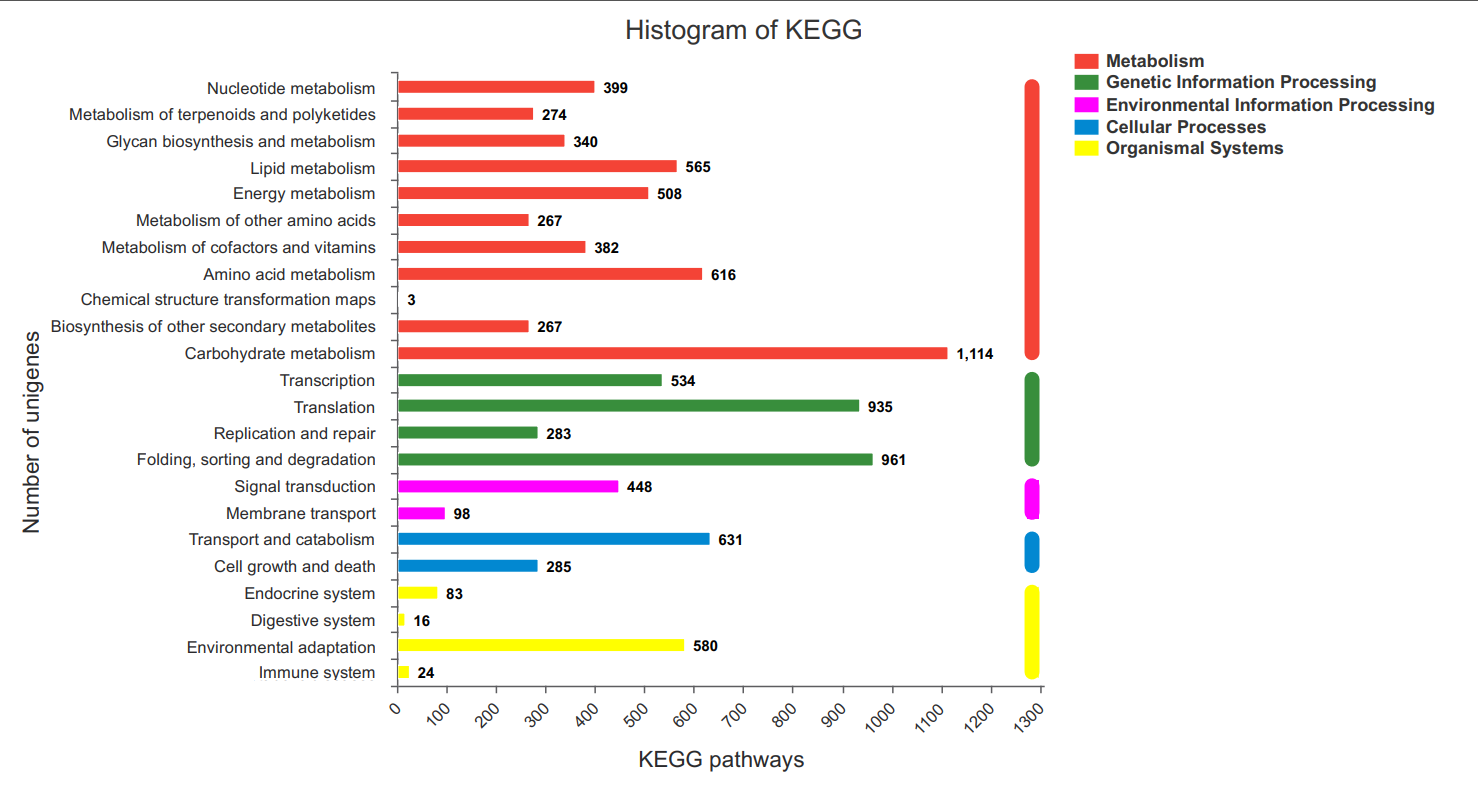

Supplement: Supplementary file 1 — Additional file 1: TableS1. Summary of RNA-Seq database from A.lancea under drought stress. Table S2. The detailed information for assembled unigenes of A. lancea under drought stress. Table S3. QRT-PCR validation of DEGs from A. lancea. Table S4. The primer list of DEGs for qRT-PCR validation. Figure S1. Functional classification for assembled unigenes of A. lancea by KEGG. [file 12870_2021_3048_MOESM1_ESM.zip › Figure S1_ESM.tif]
